# Pennsylvania State Medical Convention

**Published:** 1848-05

**Authors:** 


### THE MEDICAL EXAMINER.

### PHILADELPHIA, MAY, 1848.

### PENNSYLVANIA STATE MEDICAL CONVENTION.

In our *Record* of the present number, will be found a full account of the doings of the Convention recently held in the city of Lancaster. Having been present during a part of the time, we bear willing testimony to the courtesy and liberality displayed by its members in their discussions, and the entire absence of personal or selfish motives in any of the proceedings we were cognizant of. The members of the profession, in the city, vied with each other in kindness to their brethren from a distance, and two of the oldest and most prominent, Drs. Humes and Atlee, gave handsome entertainments, to which every member of the Convention was invited. To-morrow the National Association meets in Baltimore. From appearances, it will be largely attended by gentlemen of experience and eminence in the profession. Many interesting subjects will doubtless be presented in the reports of committees, and by the members, and if all *cliquism* be eschewed, as we hope and trust will be the case, much good may be anticipated from the comparison of views by gentlemen representing distant sections of country, and different institutions. We hope to be present during the sittings of the Association, and to be able to give an account of its proceedings in our next.

